# Efficacy of acupuncture for hypertension in the elderly: a systematic review and meta-analysis

**DOI:** 10.3389/fcvm.2023.1147135

**Published:** 2023-12-14

**Authors:** Tianyi Wang, Hangyu Li, Shixing Feng, Junqi Wang, Wanli Qin, Yuan Zhang, Wen Sun, Chenlu Wang, Xuanyi Cai, Dongran Han, Jialin Liu, Yixing Liu

**Affiliations:** ^1^ School of Management, Beijing University of Chinese Medicine, Beijing, China; ^2^ School of Life and Science, Beijing University of Chinese Medicine, Beijing, China; ^3^ Centre France Chine de la Médecine chinoise, Selles sur Cher, France; ^4^ Department of Neurology, Dongfang Hospital Beijing University of Chinese Medicine, Beijing, China; ^5^ Dongzhimen Hospital Beijing University of Chinese Medicine, Beijing, China

**Keywords:** acupuncture, hypertension, elderly, systematic review, meta-analysis

## Abstract

**Background:**

Hypertension has now developed into a major public health problem worldwide. Under the existing antihypertensive drug treatment paradigm, problems such as decreasing drug resistance and increasing drug side effects can occur for elderly patients. Acupuncture, a core technique in the non-pharmacological treatment of Chinese medicine, plays an important role in the treatment of elevated blood pressure.

**Objective:**

This study aimed to systematically evaluate the effect of acupuncture alone or in combination with antihypertensive drugs on the efficiency of reducing blood pressure and controlling blood pressure in elderly patients with hypertension.

**Methods:**

Articles of randomized controlled trials of acupuncture for hypertension in the elderly published before November 2022 were searched in 7 databases. The methodological quality of the literature was evaluated using the Cochrane Risk of Bias Assessment Tool. The primary outcome was the efficiency rate of blood pressure reduction, and the secondary outcome was the change in blood pressure after treatment.

**Results:**

This study conducted a systematic review and meta-analysis of 12 randomized controlled trials with a total of 1,466 subjects. Among the primary outcome—efficiency rate, acupuncture-only treatment (RR = 1.11, 95% CI: 1.03–1.20, *P* < 0.01) and acupuncture combined with antihypertensive drug treatment (RR = 1.18, 95% CI: 1.06–1.31, *P* < 0.01) were significantly different compared with drugs-only treatment. Among the secondary outcomes, SBP (MD: −4.85, 95% CI: −10.39 to −0.69, *P *= 0.09) and DBP (MD: −1.45, 95% CI: −5.35 to 2.45, *P* = 0.47) show no significant difference between acupuncture-only treatment and drug-only treatment. Compared to drugs-only treatment, acupuncture plus drugs has more significant efficiency in lowering SBP (MD: −9.81, 95% CI: −13.56 to −6.06, *P* < 0.01) and DBP (MD: −7.04, 95% CI: −10.83 to −3.24, *P* < 0.01).

**Conclusion:**

For elderly patients with hypertension, acupuncture-only treatment has the same efficiency and antihypertensive effect compared to drug therapy and acupuncture plus drugs outperforms drugs-only treatment. If the patients receive therapy with less frequency per week and longer duration, there will be a more obvious antihypertensive effect. Due to the methodological defects in the included study and the limited sample size of this paper, more well-designed randomized controlled trials are needed for verification.

**Systematic Review Registration:**

https://www.crd.york.ac.uk/prospero/display_record.php?ID=CRD42022376407, PROSPERO (CRD42022376407).

## 1. Introduction

Hypertension, also known as high or raised blood pressure, is a condition in which the blood vessels have persistently raised pressure (1). Several studies have shown that hypertension increases the incidence of secondary diseases, such as heart (2), brain (3), and kidney damage (4), which in turn generate additional health burdens. According to the WHO, hypertension is one of the leading causes of premature death worldwide and 1.28 billion people (by 2021) are suffering from it (1). Geriatric hypertension is a condition in which the elderly population (generally ≥60 years of age) suffers from hypertension, and an elderly hypertensive population is a special group of hypertensive patients, with characteristics mainly including predominantly elevated SBP, increased pulse pressure, large blood pressure fluctuations, early morning hypertension, postural blood pressure fluctuations, postprandial hypotension, the abnormal circadian rhythm of blood pressure, common white coat phenomenon, many complications, presence of easily missed types of hypertension (secondary hypertension, occult hypertension) and pseudohypertension.

Currently, the most widely used way for the treatment of hypertension is pharmacotherapy, which mainly includes angiotensin-converting enzyme inhibitors, angiotensin receptor blockers, calcium channel blockers, β-blockers, or thiazide diuretics. However, drug therapy has some significant drawbacks, including drug adherence problems (5) and side effects of drugs (6). A study showed that about 50% of people who were treated for hypertension had premature discontinuation of treatment (5); and for side effects of drugs, β-blockers can cause bradycardia (6). Due to the significant differences in body condition, especially in the elderly, the drug metabolism and excretion functions of the liver and kidney are bound to decrease in varying degrees. Therefore, treatment with safer and lower side effects is important for the treatment of hypertension in the elderly. Among them, non-pharmacological treatment is one of the important measures of antihypertensive therapy, emphasizing the control of blood pressure attainment and disease progression by changing bad lifestyle habits (7).

Acupuncture has been used for centuries as an important component of Chinese medicine for the treatment of elevated blood pressure in China (8) and has been widely used internationally for the treatment of cardiovascular and cerebrovascular diseases. In the 1990s, the FDA identified the use of acupuncture to treat five general conditions, including stroke (9). A bibliometric study based on the Web of Science (WoS) showed that acupuncture covers 77 diseases in 12 fields (10). In recent years, the mechanisms of acupuncture in analgesia, anti-inflammation, and neuromodulation have been the subject of a large number of mechanistic studies, providing a theoretical basis for studies related to the treatment of blood vessels with acupuncture (11–13). There are many research precedents about acupuncture in the treatment of hypertension, but there has not been a systematic review specifically for hypertension in the elderly. Hypertension in the elderly tends to have unique characteristics due to their different physical status and vulnerability from that of the young and middle-aged population. We conducted a systematic review and meta-analysis to evaluate the efficacy of acupuncture in the elderly hypertensive population.

## 2. Materials and method

This systematic review and meta-analysis followed the PRISMA statement. The review protocol was registered at PROSPERO (Registration number: CRD42022376407).

### 2.1. Search strategies

We searched the EMBASE, PubMed, Cochrane, China National Knowledge Infrastructure (CNKI), Wan Fang, VIP, and Sinomed. The following keywords combined with Medical Subject Headings (MeSH) terms were used for searching:

“Acupuncture”, “Acupuncture Treatment”, “Electroacupuncture”, “Acupuncture Therapy”, “Acupotomy”, “Auricular Acupuncture”, “Warm Needle”, “Moxibustion”, “Cardiovascular Diseases”, “cardiovascular diseases”, “High Blood Pressures”, “High Blood Pressure”, “Blood Pressures, High”, “Blood Pressure, High”, “blood pressure”, “arterial pressure”, “hypotension”, “nmotension”, “hypertensive”, “systolic pressure”, “diastolic pressure”, “pulse pressure”, “venous pressure”, “pre-hypertension”, “bp response”, “bp reduction”, “bp monit”, “bp decrease”, “bp monits”, “bp measurement”, “randomized controlled trial”, “randomized”, “RCT”.

The detailed search strategy is presented in Supplementary Material Table S1. In addition, we carefully scanned the references of randomized controlled trial articles and related reviews that met the search criteria to ensure that the literature search for meta-analyses was complete. No language restrictions were applied. Data collection was completed in November 2022 and we searched documents in the databases up to that point.

### 2.2. Eligibility criteria

Two authors (JQ WANG and WL QIN) independently screened the eligible clinical trials based on the criteria as follows:

Inclusion criteria: (1) A randomized controlled trial of acupuncture for hypertension; (2) Patients diagnosed with hypertension with systolic blood pressure (SBP) ≥140 mmHg and/or diastolic blood pressure (DBP) ≥90 mmHg or on anti-hypertensive medication; (3) Participants ≥60 years of age; (4) Patients in the intervention group received treatment including acupuncture (electroacupuncture, auricular acupuncture, warm acupuncture, dry acupuncture, auricular pressure beans) more than once, with or without antihypertensive drugs. (5) The control group was treated with antihypertensive drugs; (6) The efficiency rate of treatment, and the change in SBP and DBP after treatment compared to the baseline were included in the results of the study.

Exclusion criteria: (1) Duplicate literature in the library; (2) Case studies, animal experiments, and ethical explorations; (3) Studies with only a summary but no specific data; (4) Non-RCT trials, semi-randomized controlled trials, and self-controlled trials; (5) Involving other forms of acupuncture, such as transcutaneous electrical nerve stimulation, laser acupuncture; (6) Unable to convert blood pressure data units to mmHg.

According to the inclusion and exclusion criteria, two authors (W Sun and CL Wang) independently searched the databases to obtain eligible clinical trials. One author (W Sun) read the full text of the eligible collected articles. Another author (CL Wang) checked the accuracy and completeness of the collected articles. During the process of study selection, two authors solved the disagreements through discussion. If consensus is not reached, a third author (Y Zhang) will step in to discuss resolved differences.

### 2.3. Data items

Two authors (W Sun and XY Cai) independently used Excel software to extract the data. Data included: first author, date of publication, number of patients, mean age of patients, acupuncture treatment method, acupuncture points, frequency of acupuncture, acupuncture treatment duration, type of control group, mean blood pressure before treatment, mean blood pressure after treatment, mean blood pressure change, treatment efficiency, and adverse events. Missing data or information was requested from the lead author by email or phone if necessary. If a consensus is not reached, a third author (SX Feng) will make the final decision.

### 2.4. Types of outcomes measurements

The primary outcome of this study was the efficiency rate of treatment, defined as a 10 mmHg decrease in SBP or a 5 mmHg decrease in DBP. The secondary outcome was the change in blood pressure after treatment (change in blood pressure = previously treated blood pressure value—post-treatment blood pressure value).

### 2.5. Quality assessment

According to the RoB assessment tool in the Cochrane Handbook (14), each included study was evaluated independently by two authors (JQ Wang and WL Qin), including the risk of bias in the following aspects: (1) Random sequence generation; (2) Allocation concealment; (3) Blinding of participants and personnel; (4) Blinding of outcome assessment; (5) Incomplete outcome data; (6) Selective reporting; (7) Other bias. Based on the Cochrane Assessment Tool, our judgments for these domains were categorized as “low risk of bias”, “high risk of bias”, or “unclear risk of bias”. Disagreements between two authors in the evaluation are resolved through discussion, and when consensus cannot be reached, a decision is made by a third author (SX Feng).

### 2.6. Statistics analysis

In this study, the R 4.2.1 software, and the Review Manager version 5.3 software (Cochrane Collaboration, Nordic Cochrane Centre, Copenhagen, Denmark) were employed to analyze the collected data.

The R 4.2.1 software was used for the statistical analysis. The dichotomous variable was represented as the pooled risk ratios (RRs) with 95% CI. The continuous variable was represented as the mean difference (MD) with 95% CI. *I*^2^ statistics and the chi-squared test were applied to evaluate the heterogeneity among the included RCTs. If *I*^2^ > 50% or *P* < 0.1, it suggested that a significant statistical heterogeneity was observed, and the random-effect model should be employed to evaluate the outcome measures. Otherwise, the fixed-effect model was adopted. If *P* < 0.05, it suggested that there was a significant statistical difference in this meta-analysis.

We performed subgroup analyses of changes in treatment frequency and duration to assess the effects of different acupuncture treatment protocols. Where feasible, sensitivity analyses of the results were performed to explore the robustness of the summary findings.

The funnel plot created by the R 4.2.1 software was employed to evaluate the potential publication bias. Peter's test and Egger's test were also used to detect the potential publication bias of the categorical variable and the continuous variable in this meta-analysis respectively.

## 3. Results

### 3.1. Study selection

**Figure 1:**
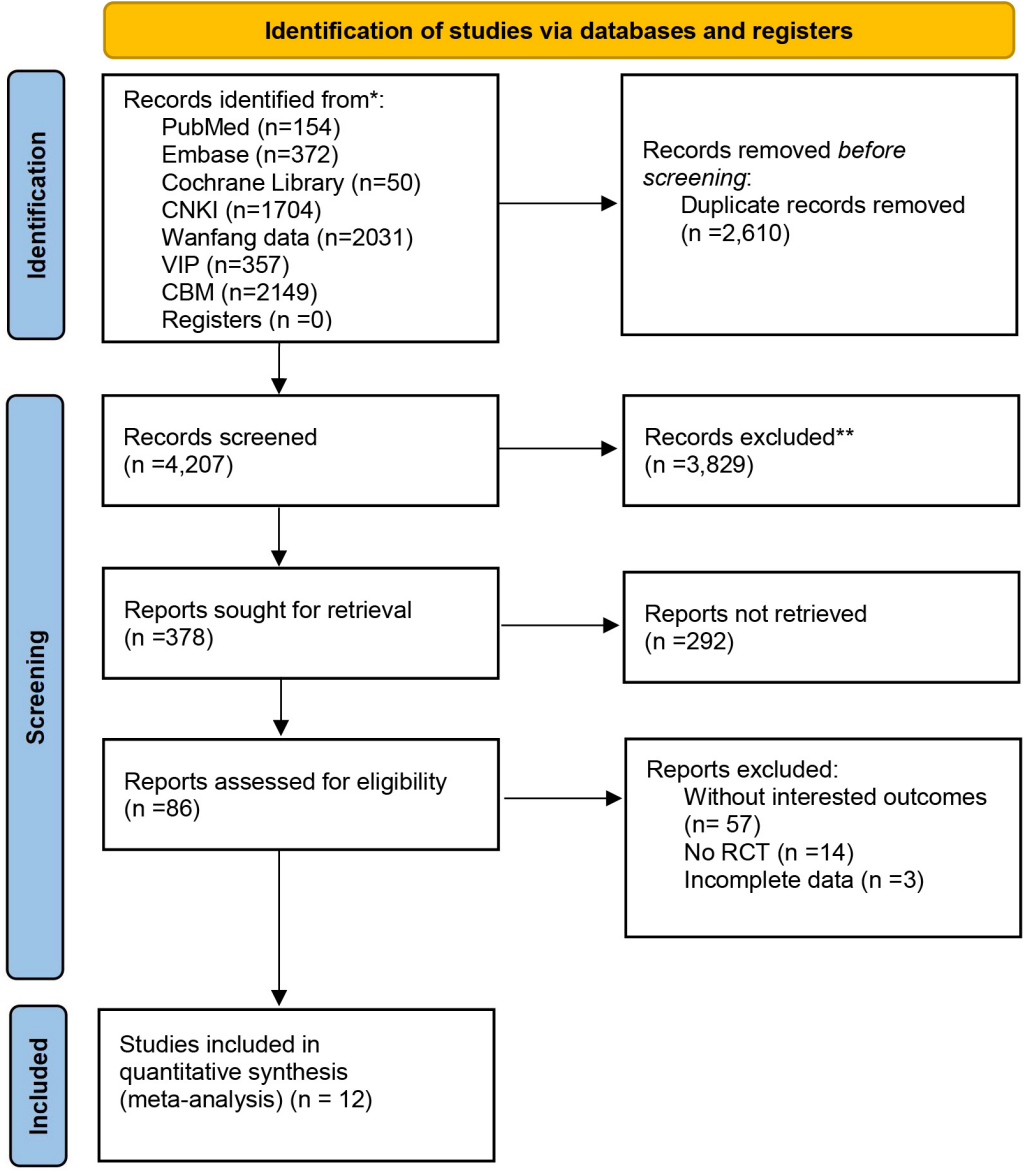
PRISMA flow diagram of the trial selection process.

The process of study selection for the eligible RCTs is shown in Figure 1. A total of 6,817 potential studies were preliminarily identified from the database based on the search strategy, 6,439 of which were excluded due to review or duplication. We conducted a secondary search of the remaining 378 documents, with 292 removed, and further screened the remaining 86 documents. Finally, after excluding studies that did not include relevant outcome indicators, non-RCT studies, and studies with incomplete data, a total of 12 randomized controlled trials were included for further quality assessment and meta-analysis. The detailed process of identification and selection is shown in the PRISMA flow diagram (15).

### 3.2. Study characteristics

The meta-analysis included a total of 12 RCT studies (16–27) from 2007 to 2022, covering 1,466 participants in Table 1 (the minimum inclusion number was 60 participants and the maximum was 236). These participants were recruited in hospital outpatient and inpatient units as well as in the community, with an average age of 60 years or older. For the acupuncture methods used in each study, six papers used general acupuncture while the other six used acupuncture combined with auricular acupuncture or auricular point pressing. The most used acupuncture point was Quchi, which was mentioned in nine studies, followed by Sanyinjiao (8), Baihui (8), Hegu (8), and Zusanli (7). The frequency of weekly interventions ranged from once a week to seven times a week; the duration of treatment ranged from 1 week to 12 weeks. Among the 4 of 12 RCTs, the intervention groups were treated with acupuncture, while the control groups were treated with drugs; in the other eight trials, the intervention groups were treated with acupuncture combined with drugs, and the control groups were treated with drugs.

**Table 1:** The characteristics of included studies in the meta-analysis.

Author (year)	Participants	Mean age		Intervention^a^		Duration	Frequency	Acupuncture methods	Outcome^b^	Acupuncture points	Drug for the trials	
		Treatment	Control	Treatment	Control						Treatment	Control
Zheng et al. (16)	60	61.27	63.43	AC	CA	8 weeks	Twice a week	Yes	1/2/3	DU20, RN12, RN6, LI11, ST36, SP6, LR3, GB20, PC6, BL15, BL17, BL23	–	Nifedipine controlled release tablets
Hao et al. (17)	60	64.57	64.77	AC	ACEI	1 weeks	Once a week	Yes	1/2	LR3	–	Captopril tablets
Dou (18)	200	66.79	65.79	AC	Drug	12 weeks	Three times a week	Yes	1/2	LR3, LI11, HT7, DU20, LI4, ST36, SP6	–	Common drug for anti-hypertension
Shao (19)	100	72.11	74.13	AC + AP	Drug	12 weeks	Three or four times a week	Yes	1/2	GB20, LI11, DU14, ST36, LR3, PC6, LI4, DU20, EX-HN5, SP6	–	Common drug
Liu C and Liu Y (20)	60	78.64	78.62	AC + AP + AngII	AngII	8 weeks	Once every two days	Yes	1/2	ST36, DU20, HT7, LI11, LR3, LI4	Irbesartan	Irbesartan
Jin (21)	124	73.24	71.36	AC + AP + Drug	Drug	10 weeks	Three times a week	Yes	1/2	SP6, DU20, ST36, LI11, GB20, LR3, HT7, LI4	Common drug	Common drug
Zhao (22)	180	70.03	69.68	AC + CA	CA	12weeks	Once every two days	Yes	1/2	LI4	Amlodipine besylate tablets	Amlodipine besylate tablets
Che (23)	110	62.4	61.9	AC + AngII	AngII	4 weeks	Once a week	Yes	1/2/3	ST9, LI4, LR3, LI11, ST36,	Irbesartan	Irbesartan
Hu et al. (24)	60	77.80	77.10	AC + CA	CA	3 weeks	Once a day for three weeks	Yes	1/2	DU20, GB20, LI11, SP6	Amlodipine	Amlodipine
Li et al. (25)	104	67.00	67.00	AC + ACEI	ACEI	12 weeks	Five times a week	Yes	1/2	LI4, DU20, KI3, LR3, SP6, LI11	Enalapril maleate tablets	Enalapril maleate tablets
Ye (26)	236	61.80	60.35	AC + Drug	Drug	10 weeks	Three times a week	Yes	1/2	GB20, LI11, SP6, LI4, LR3, ST36, DU20, HT7	Common drug	Common drug
Huang J and Huang X (27)	80	71.82	72.26	AC + CA	CA	4 weeks	Six times a week	Yes	1/2/3	ST37, ST40	Nifedipine delayed-release tablets	Nifedipine delayed-release tablets

^a^ AC, acupuncture; CA, calcium antagonists; Drug, only drug mentioned; ACEI, ACE inhibitor; AngII, angiotensin II; AP, auricular pressure.

^b^ 1, SBP/DBP value before/after treatment; 2, response rate of treatment; 3, adverse reactions.

In all trials, the efficiency rate was selected as the primary outcome, and the change in mean systolic and diastolic blood pressure before and after the trials serves as the secondary outcome. Except for one trial (23) lacking the secondary outcome, all studies reported complete primary and secondary outcomes.

### 3.3. Risk of bias in individual studies

**Figure 2:**
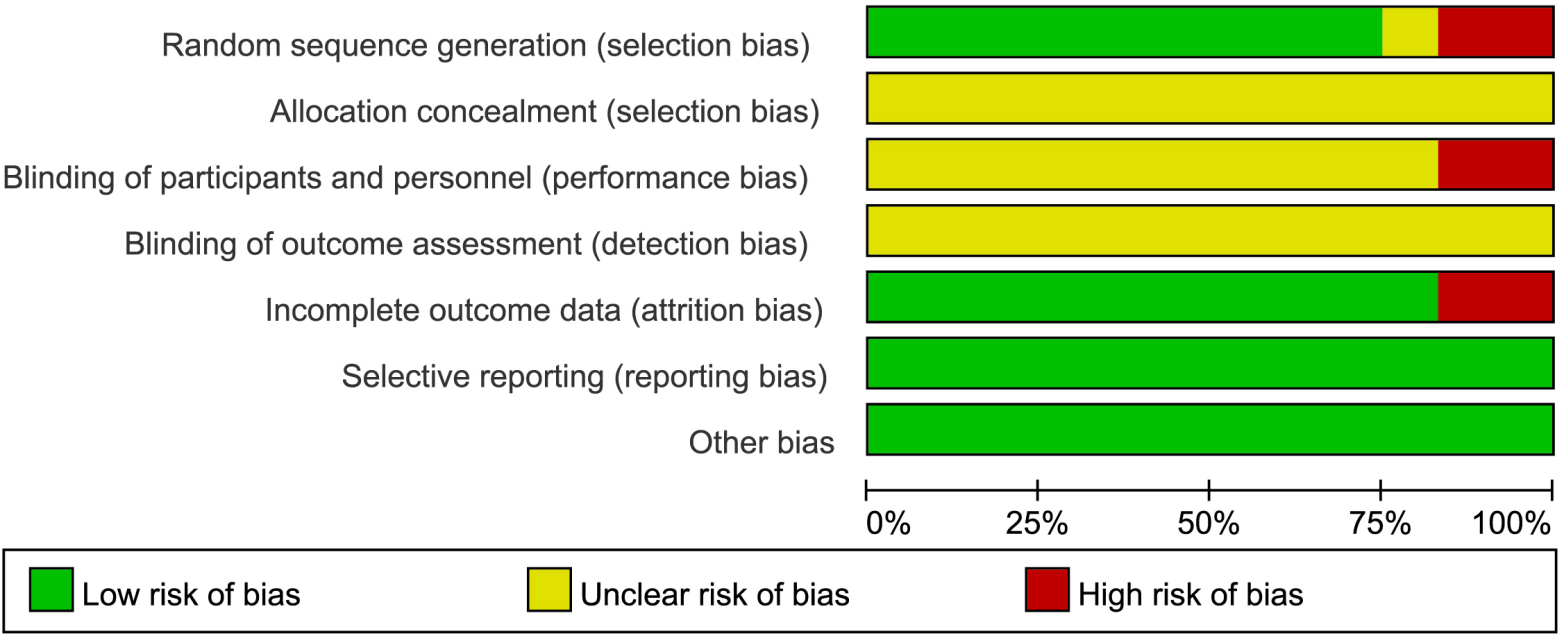
Risk of bias summary: review authors’ judgments about each risk of bias item for each included study.

**Figure 3:**
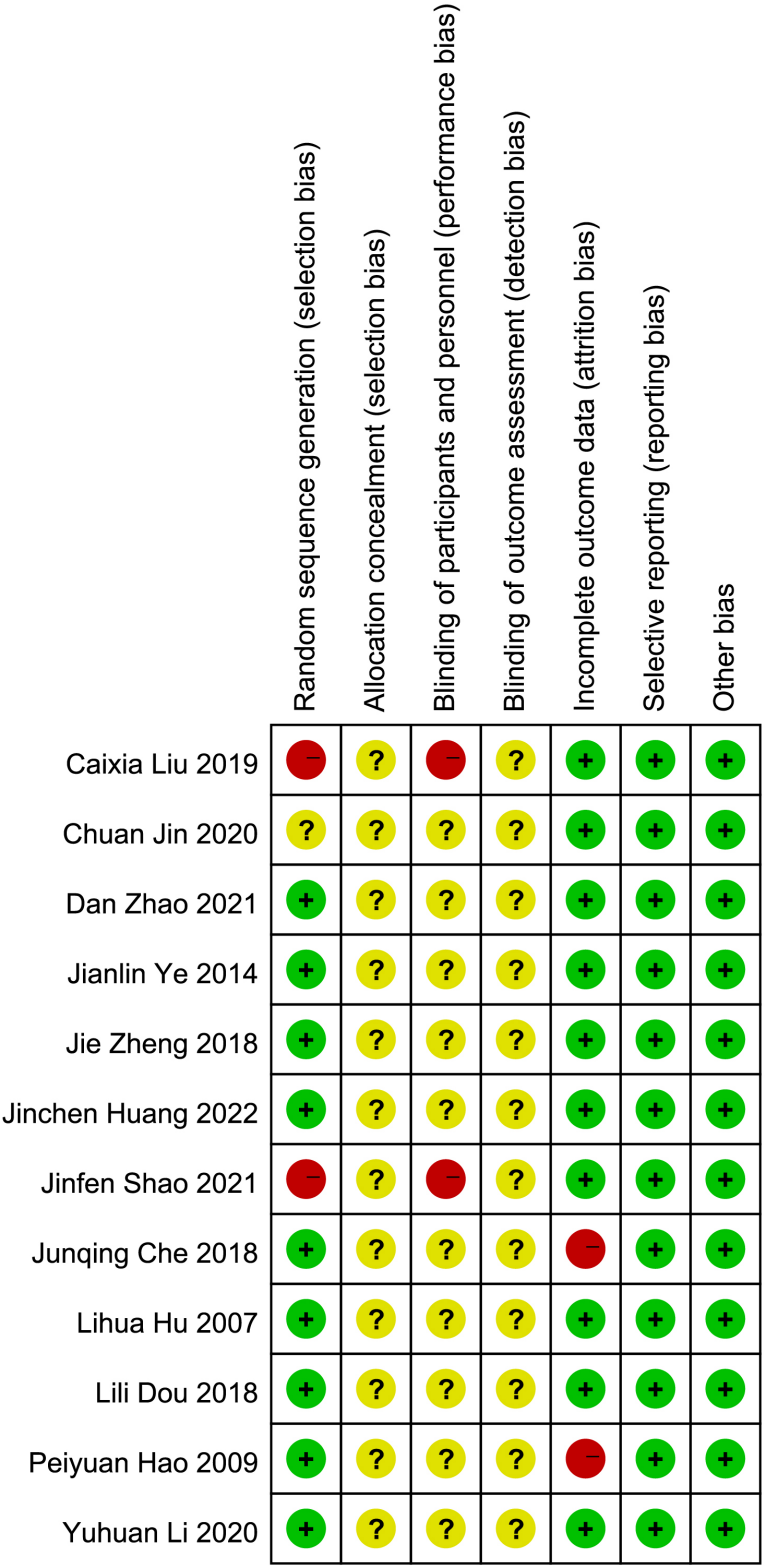
The risk of bias for the included studies.

We summarize the risk of bias assessment for each of the included studies in Figures 2, 3.

#### 3.3.1. Random sequence generation

Nine studies (16–18, 22–27) had a low risk of randomization bias, which were described as RCTs and indicated that randomization grouping was performed. One (21) study did not mention the grouping method, which we assessed as “unknown”. Two (19, 20) studies were grouped according to treatment and therefore assessed as “high risk”.

#### 3.3.2. Allocation concealment

None of the 12 studies (16–27) we included mentioned randomized concealment and were all assessed as “unknown”.

#### 3.3.3. Blinding of participants and personnel and outcome assessment

Two studies (19, 20) were assessed as high risk for blinding due to the subjects' choice of treatment according to their individual wishes. The remaining 10 studies (16–18, 21–27) were assessed as “unknown”. In addition, all trials (16–27) were rated as having an unclear risk of assay bias because no blinding of clinicians, analysts, or data collectors was reported.

#### 3.3.4. Incomplete outcome data

Ten studies (16, 18–22, 24–27) were assessed as low risk for outcome reporting, and two studies (17, 23) were rated as high risk due to incomplete endpoint data as a result of subject withdrawal.

#### 3.3.5. Selective reporting

Although all studies (16–27) did not have a trial protocol, reported planned outcomes in the study and therefore we judged them to be low risk.

#### 3.3.6. Other potential sources

No other factors affecting bias were mentioned in any of the 12 studies (16–27), so we judged it to be low risk.

### 3.4. Acupuncture vs. drug-only treatment

#### 3.4.1. Efficiency rate

**Figure 4:**
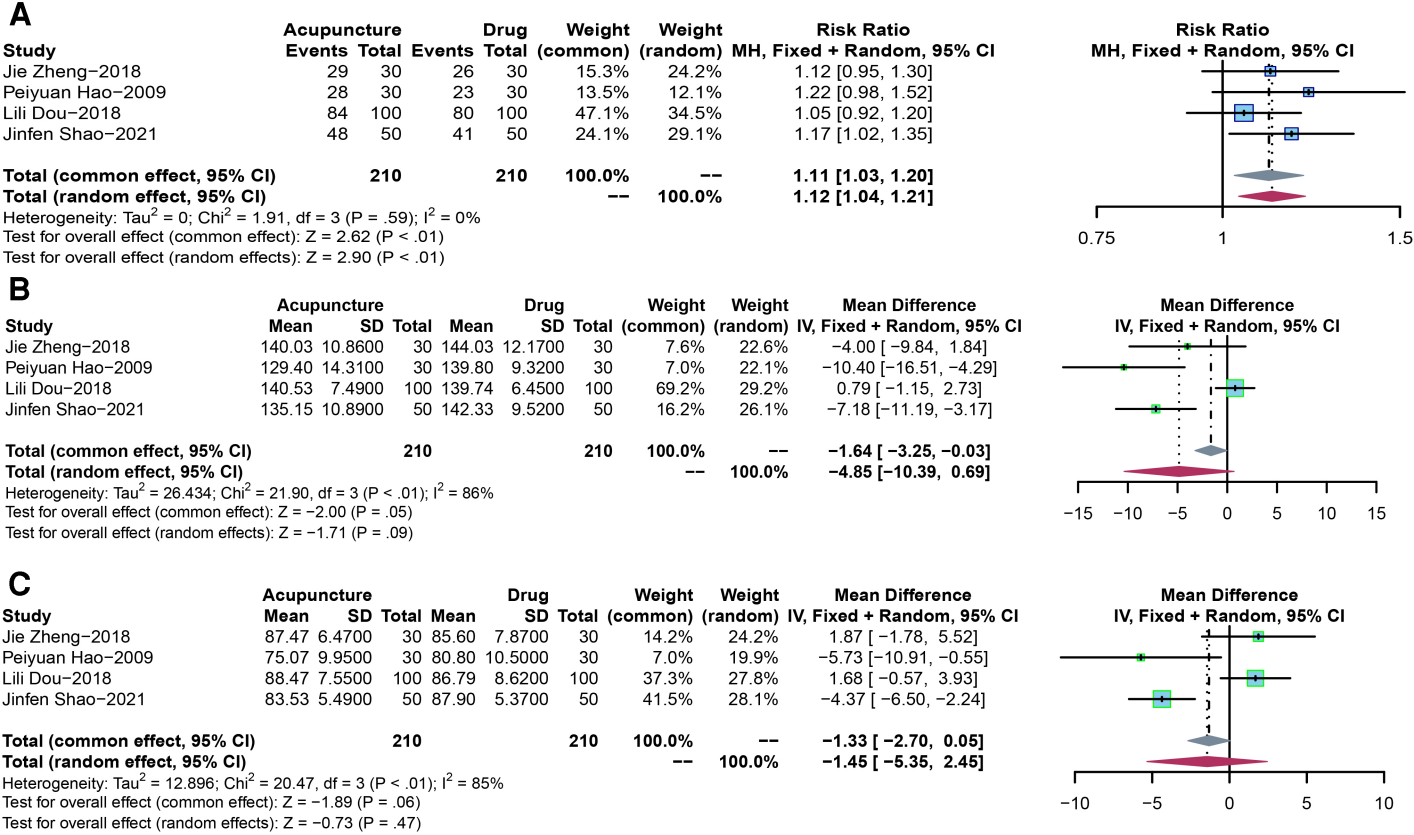
(**A**) The forest plot shows the effect of acupuncture vs. drug treatment on efficiency in the elderly. (**B**) The forest plot shows the effect of acupuncture vs. drug treatment on SBP in the elderly. (**C**) The forest plot shows the effect of acupuncture vs. drug treatment on DBP in the elderly.

In order to compare the efficacy of acupuncture and drugs in lowing blood pressure in the elderly population, four RCTs including 420 subjects were analyzed to assess the efficiency. Due to the presence of low heterogeneity (*P* = 0.59, *I*^2 ^= 0%), a fixed-effect model was used. The results showed that acupuncture, compared with drug treatment, had a significant difference in the antihypertensive efficiency rate (RR = 1.11, 95% CI: 1.03–1.20, *P* < 0.01) (Figure 4A).

#### 3.4.2. Systolic blood pressure (SBP)

Four RCTs with 420 subjects were analyzed to assess changes in SBP. A random-effect model was used due to the high heterogeneity (*P* < 0.01, *I*^2^ = 86%). The results showed no significant difference in lowering SBP between acupuncture and drugs (MD: −4.85, 95% CI: −10.39 to −0.69, *P* = 0.09). We had to cancel the subgroup analysis because of the small number of included literature, with only 4 papers covered (Figure 4B).

#### 3.4.3. Diastolic blood pressure (DBP)

We analyzed four RCTs covering 420 participants to assess changes in DBP. Given the high heterogeneity (*P* < 0.01, *I*^2^ = 85%), We adopted a random-effect model. The results showed no significant difference in lowering DBP between acupuncture and drugs (MD: −1.45, 95% CI: −5.35 to 2.45, *P* = 0.47). We had to cancel the subgroup analysis because of the small number of included literature, with only 4 papers covered (Figure 4C).

### 3.5. Acupuncture combined with drug vs. drug-only treatment

#### 3.5.1. Efficiency rate

##### 3.5.1.1. Meta-analysis

**Figure 5:**
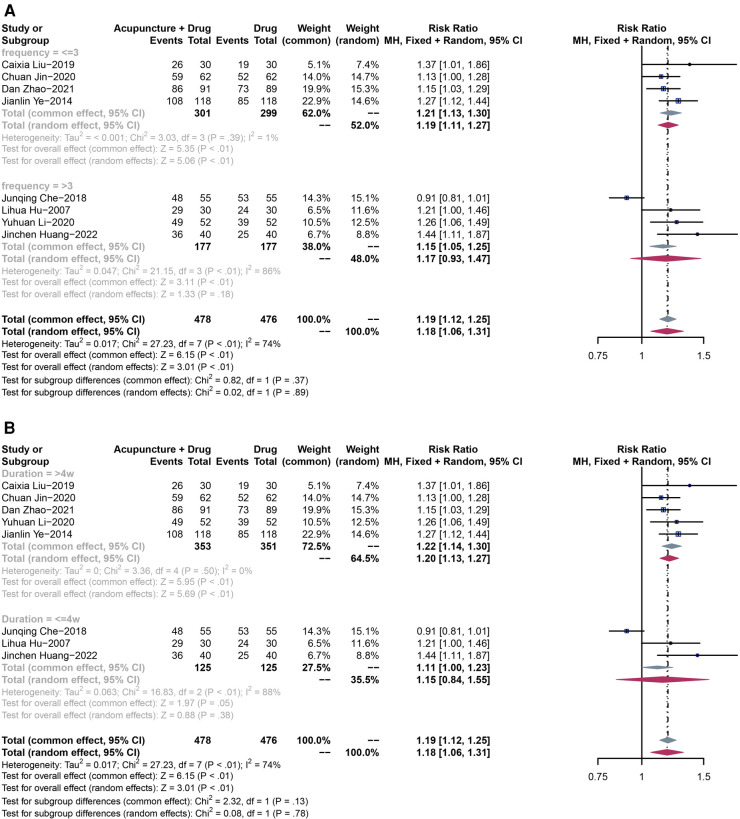
(**A**) The forest plot shows the effect of acupuncture combined with drugs vs. drugs-only treatment on the efficiency rate in subgroups stratified according to treatment frequency. (**B**) The forest plot shows the effect of acupuncture combined with drugs vs. drugs-only treatment on the efficiency rate in subgroups stratified according to the treatment duration.

In order to compare the efficacy of acupuncture combined with drugs vs. drug-only treatment in lowing blood pressure in the elderly, eight RCTs including 954 patients were analyzed to assess the efficiency. Due to the high heterogeneity (*P* < 0.01, *I*^2^ = 74%), a random-effect model was used. The results showed that acupuncture combined with drug, compared with drug-only treatment, had a significant difference in lowering blood pressure (RR = 1.18, 95% CI: 1.06–1.31, *P* < 0.01) (Figure 5).

##### 3.5.1.2. Subgroup analysis

Given the high heterogeneity in the efficiency rate (*P* < 0.01, *I*^2 ^= 74%), we conducted subgroup analyses based on treatment frequency and duration, to clarify the source of heterogeneity, providing more valuable evidence for clinical practice. The same subgroup analysis method was used for all the following outcomes.

We divided participants into two subgroups based on treatment frequency (subgroup 1: frequency ≤3 times; subgroup 2: frequency >3 times). Due to the low heterogeneity in subgroup 1 (*P* = 0.39, *I*^2 ^= 1%), a fixed-effect model was used. Acupuncture combined with drugs showed a significant difference in lowering blood pressure in the elderly when treatment frequency is less than three times a week (RR = 1.21, 95% CI: 1.13–1.30, *P* < 0.01), compared with drugs-only treatment. On the other hand, subgroup 2 had a high heterogeneity (*P* < 0.01, *I*^2 ^= 86%), therefore, a random-effect model was adopted. In this subgroup, the results showed no significant difference between the two groups (RR = 1.17, 95% CI: 0.93–1.47, *P* = 0.18) (Figure 5A).

We also divided participants into two subgroups based on treatment duration (subgroup 1: duration >4 weeks; subgroup 2: duration ≤4 weeks). Due to the low heterogeneity in subgroup 1 (*P* = 0.50, *I*^2 ^= 0%), a fixed-effect model was used. Acupuncture combined with drugs showed a significant difference in lowering blood pressure in the elderly when the treatment duration is more than four weeks (RR = 1.22, 95% CI: 1.14–1.30, *P* < 0.01), compared with drugs-only treatment. On the other hand, subgroup 2 had a high heterogeneity (*P* < 0.01, *I*^2 ^= 88%), therefore, a random-effect model was adopted. In this subgroup, the results showed no significant difference between the two groups (RR = 1.15, 95% CI: 0.84–1.55, *P* = 0.38) (Figure 5B).

#### 3.5.2. SBP

##### 3.5.2.1. Meta-analysis

**Figure 6:**
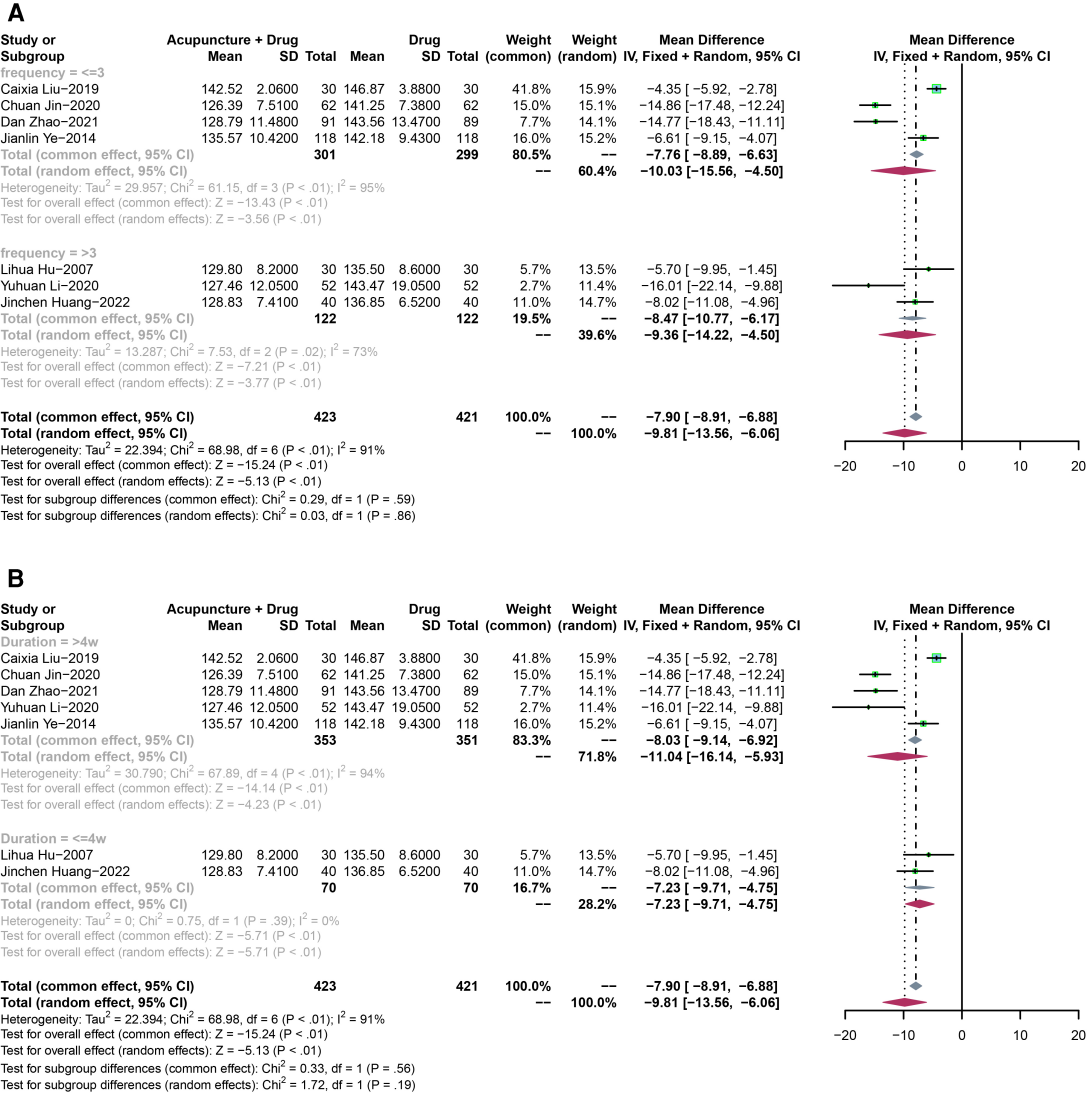
(**A**) The forest plot shows the effect of acupuncture combined with drugs vs. drugs-only treatment on the SBP in subgroups stratified according to treatment frequency. (**B**) The forest plot shows the effect of acupuncture combined with drugs vs. drugs-only treatment on the SBP in subgroups stratified according to the treatment duration.

Seven RCTs with 844 participants were analyzed to assess changes in SBP. A random-effect model was used due to the high heterogeneity (*P* < 0.01, *I*^2^ = 91%). The results showed a significant difference in lowering SBP between acupuncture plus drugs and drugs-only treatment (MD: −9.81, 95% CI: −13.56 to −6.06, *P* < 0.01) (Figure 6).

##### 3.5.2.2. Subgroup analysis

Pooled SBP showed a high heterogeneity (*P* < 0.01, *I*^2^ = 91%).

In the subgroup analysis according to treatment frequency, both of the results for frequency less than three times a week (MD: −10.03, 95% CI: −15.56 to −4.50, *P* < 0.01) and frequency more than three times a week (MD: −9.36, 95% CI: −14.22 to −4.50, *P* < 0.01)) showed that acupuncture plus drugs had a significant difference compared with drugs-only treatment (Figure 6A).

For the treatment duration, both duration less than 4 weeks (MD: −7.23, 95% CI: −9.71 to −4.75, *P* < 0.01) and more than 4 weeks (MD: −11.04, 95% CI: −16.14 to −5.93, *P* < 0.01) showed that acupuncture plus drugs had a significant difference compared with drugs-only treatment (Figure 6B).

#### 3.5.3. DBP

##### 3.5.3.1. Meta-analysis

**Figure 7:**
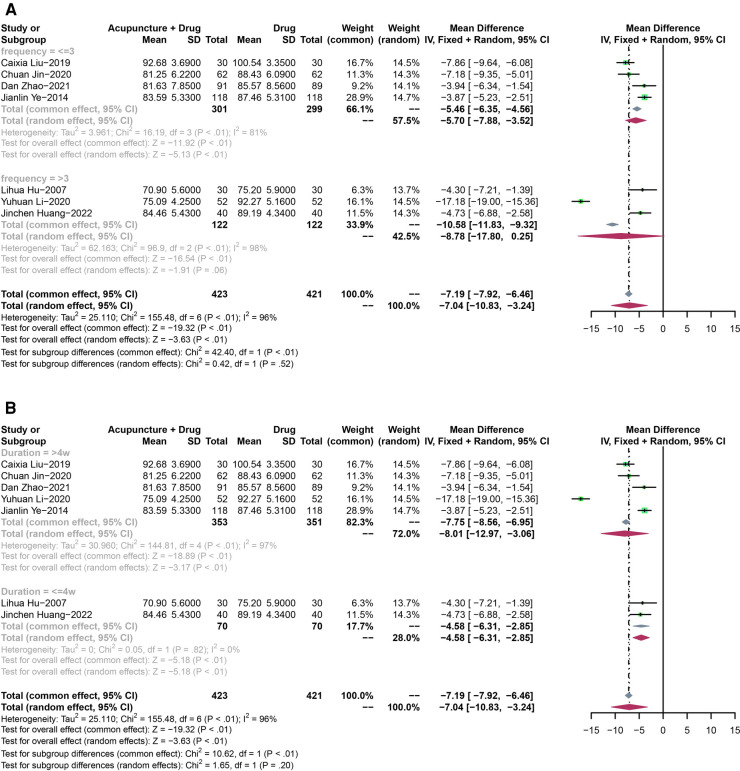
(**A**) The forest plot shows the effect of acupuncture combined with drugs vs. drugs-only treatment on the DBP in subgroups stratified according to treatment frequency. (**B**) The forest plot shows the effect of acupuncture combined with drugs vs. drugs-only treatment on the DBP in subgroups stratified according to the treatment duration.

Seven RCTs with 844 participants were analyzed to assess changes in DBP. A random-effect model was used due to the high heterogeneity (*P* < 0.01, *I*^2^ = 96%). The results showed a significant difference in lowering DBP between acupuncture plus drugs and drugs-only treatment (MD: −7.04, 95% CI: −10.83 to −3.24, *P* < 0.01) (Figure 7).

##### 3.5.3.2. Subgroup analysis

Pooled DBP showed a high heterogeneity (*P* < 0.01, *I*^2^ = 96%).

In the subgroup analysis, both of the results for frequency less than three times a week (MD: −10.03, 95% CI: −15.56 to −4.50, *P* < 0.01) and frequency more than three times a week (MD: −9.36, 95% CI: −14.22 to −4.50, *P* < 0.01)) showed that acupuncture plus drugs had a significant difference compared with drugs-only treatment (Figure 7A).

For the treatment duration, both of the results for less than 4 weeks (MD: −7.23, 95% CI: −9.71 to −4.75, *P* < 0.01) and more than 4 weeks (MD: −11.04, 95% CI: −16.14 to −5.93, *P* < 0.01) showed that acupuncture plus drugs had a significant difference compared with drugs-only treatment (Figure 7B).

## 4. Discussion

### 4.1. Summary of findings

In this study, a systematic review and meta-analysis of 12 RCTs of acupuncture for hypertension in the elderly were conducted, covering 1,466 participants. We selected the efficiency rate and changes in mean SBP and DBP as primary and secondary outcomes. First, acupuncture alone or combined with drugs outperforms drugs-only treatment, as evidenced by an improvement in blood pressure values after the interventions.

Secondly, we conducted subgroup analyses based on treatment frequency and duration. The results showed that acupuncture alone or combined with drugs, at less treatment frequency or longer treatment duration, has a positive impact on increasing the efficiency rate and lowering SBP and DBP. However, the clinical evidence is still relatively insufficient and further research is needed.

Finally, studies on side effects do not allow for an adequate evaluation of the safety of acupuncture because of the small amount of literature mentioned.

### 4.2. Mechanisms of acupuncture

A large number of clinical studies have demonstrated that acupuncture can be used as an effective method for controlling hypertension (28, 29). A randomized controlled trial found a reduction in both systolic blood pressure (SBP) and diastolic blood pressure (DBP) after 6 weeks of biweekly acupuncture treatment compared to the sham acupuncture group (30). In addition, acupuncture reduced aortic SBP and improved arterial stiffness and reflection waves in middle-aged and elderly patients with hypertension (31). Some evidence suggests that acupuncture can affect RAAS as well as the central sympathetic and endocrine systems (32). Theoretically, acupuncture reduces reflex hypertension by regulating the activity of precardiovascular sympathetic neurons on the ventrolateral (RVLM) end of the medulla oblongata. In addition to this, acupuncture was able to inhibit the activation of neurons in the arcuate nucleus of the hypothalamus, the ventral lateral nucleus of the gray nucleus around the midbrain aqueduct, and the pallid nucleus of the medulla oblongata, resulting in decreased activity of premotor sympathetic neurons in the ventral lateral aspect of the medulla oblongata head (RVLM).

Acupuncture can cause changes in plasma levels of serotonin, aldosterone, angiotensin II, renin, and norepinephrine (33). Previous studies have shown that acupuncture plays a role in lowering blood pressure through its effects on the nervous system (including afferent pathways, central nervous system, and efferent pathways) and neurotransmitters. Yang M (34) compared the levels of 47 compounds that have been reported in the literature in plasma before and after patients received acupuncture by a metabolomic approach to screen for compounds that may be relevant to the lowering of blood pressure by acupuncture. Finally, they found that there were significant differences in the levels of oleic acid and inositol before and after acupuncture and that acupuncture reduced the plasma levels of these two compounds in hypertensive patients, and inferred that they may be related to the mechanism of blood pressure lowering by acupuncture. Oleic acid can regulate monounsaturated fatty acid content and ultimately adrenergic *α* and *β* receptor efficacy by altering cell membrane lipid structure as well as α2-adrenergic receptor pathways, which in turn affects central and peripheral blood pressure levels (35). Acupuncture regulates inositol (an insulin-like characteristic protein with insulin-sensitizing properties) levels probably by improving vascular smooth muscle diastolic capacity and affecting blood pressure (36, 37). Generally speaking, serving as a non-pharmacological intervention, acupuncture has been used by clinicians to improve and treat a variety of cardiovascular diseases and also has the advantage of reducing treatment costs, adverse effects, and complications (38). Therefore, as a safe and effective adjuvant therapy for hypertension, acupuncture has been widely used in clinics, and has a significant effect on reducing blood pressure.

### 4.3. Strengths and limitations

This study, differing from other studies that set all adults with hypertension as the study population, analyzes the elderly hypertensive population specifically. We found that acupuncture-only treatment had the same antihypertensive effect compared with drugs and that acupuncture plus drugs was more effective than using antihypertensive drugs alone. What's more, our research analyzed the effect of acupuncture from other perspectives, such as the treatment frequency and duration, providing a more detailed treatment plan for acupuncture as an adjunct to antihypertensive medication.

There are still several limitations of this study: (1) The randomized controlled trials included in this study have low methodological quality, and some articles did not specify the blind method and specific intervention measures, which could potentially lead to errors in the results of the meta-analysis; (2) Few studies were conducted in this area, with only 12 RCTs included, which may have led to publication bias and low-certainty evidence for the results; (3) All trials were conducted in China, so it may be difficult to generalize the findings to other countries, especially those with different views and beliefs about acupuncture, which may lead to publication bias; (4) In this meta-analysis, the observed benefit correlated only with the outcome at the end of treatment. The long-term effects of acupuncture, alone or in combination with antihypertensive therapy, in the treatment of hypertension in older adults are unclear and worthy of further study.

### 4.4. Implications

This systematic review and meta-analysis may have some potential implications for clinical practice. Firstly, the use of acupuncture plus antihypertensive drugs outperforms drugs-only treatment in the elderly with hypertension, which has important practical clinical value in terms of improving remission rates as well as reducing the number of elderly people using antihypertensive drugs. Therefore, based on our research, we recommend that elderly patients using antihypertensive medications, especially those who have responded poorly or have adverse effects to antihypertensive medications, also receive acupuncture treatment. Meanwhile, when acupuncture treatments are performed, low-frequency, long-term treatment will have a more pronounced effect on lowering blood pressure. Usually, the acupuncturist will set an individualized acupuncture plan for each patient based on the patient's symptoms and TCM theory, but the randomized controlled trials included in this study showed differences in acupoint selection, type of acupuncture, treatment duration, and frequency. Thus, the optimal prescription of acupuncture for elderly patients with hypertension remains unclear.

Hypertension is an important risk factor for cardiovascular disease, and its greatest disease burden is that it can damage the function of essential organs such as the heart, brain, and kidneys, eventually leading to the failure of these organs. Therefore, to confirm the evaluation of the efficacy of acupuncture in elderly patients with hypertension, more attention should be paid to endpoint events, such as distant-related cardiovascular events and target organ damage, in addition to the observation of blood pressure values. Future work should combine high-quality and large-scale randomized controlled trials with meta-analyses to characterize the effectiveness and safety of acupuncture in the treatment of elderly patients with hypertension and to determine the best treatment options.

## 5. Conclusion

In conclusion, our systematic review and meta-analysis found that acupuncture-only treatment had the same efficacy and antihypertensive effect compared with drugs, and acupuncture plus drugs outperforms drugs-only treatment. Besides, when acupuncture treatments are performed, low-frequency, long-term treatment will have a more pronounced effect on lowering blood pressure, which can improve remission rates as well as reduce the number of elderly people using antihypertensive drugs. The results of our meta-analysis should be considered with caution due to the methodological shortcomings of the included studies and the limited sample size of this paper. In future clinical studies, attention should be paid to the standardized and scientific design required for randomized controlled trials, and studies with multicenter, large samples and sufficient follow-up time should be conducted in order to derive more systematic objective, and accurate curative effect, provide reliable evidence for further proving the superiority of long-term efficacy of acupuncture in the treatment of hypertension in the elderly.

## Data Availability

The original contributions presented in the study are included in the article/**Supplementary Material**, further inquiries can be directed to the corresponding authors.
