# Supplementary material for: Efficacy of acupuncture for hypertension in the elderly: a systematic review and meta-analysis
Source: Front Cardiovasc Med. 2023 Dec 14;10:1147135. doi: 10.3389/fcvm.2023.1147135 (PMC10756235; doi:10.3389/fcvm.2023.1147135)
Supplement: Supplementary file 1 [file Table1.docx]

**Search Strategies**

The search was conducted using a combination of subject terms and free terms, and was adjusted to the characteristics of each database. References included in the study were also searched to supplement access to relevant information. Search terms include: Acupuncture, Acupuncture Treatment, Hypertension, Blood Pressure, Randomized Controlled Trial etc. Take PubMed as an example, its specific search strategy is:

| #1 Acupuncture [MeSH Terms] |
| --- |
| #2 Acupuncture [Title/Abstract] |
| #3 Acupuncture Treatment[MeSH Terms] |
| #4 Acupuncture Treatment[Title/Abstract] |
| #5 Acupuncture Therapy[MeSH Terms] |
| #6 Acupuncture Therapy[Title/Abstract] |
| #7 Acupuncture Therapies[MeSH Terms] |
| #8 Acupuncture Therapies[Title/Abstract] |
| #9 Acupotomy[MeSH Terms] |
| #10 Acupotomy[Title/Abstract] |
| #11 Dry-needling[MeSH Terms] |
| #12 Dry-needling[Title/Abstract] |
| #13 Electroacupuncture[MeSH Terms] |
| #14 Electroacupuncture[Title/Abstract] |
| #15 Warm Needle[MeSH Terms] |
| #16 Warm Needle[Title/Abstract] |
| #17 Moxibustion [MeSH Terms] |
| #18 Moxibustion [Title/Abstract] |
| #19 Auricular Acupuncture [MeSH Terms] |
| #20Auricular Acupuncture [Title/Abstract] |
| #21 #1 OR #2 OR #3 OR #4 OR #5 OR #6 OR #7 OR #8 OR #9 OR #10 OR #11 OR #12 OR #13 OR #14 OR #15 OR #16 OR#17 OR#18 OR #19 OR #20 |
| #22Hypertension[MeSH Terms] |
| #23Hypertension[Title/Abstract] |
| #24Cardiovascular Diseases[MeSH Terms] |
| #25Cardiovascular Diseases[Title/Abstract] |
| #26cardiovascular diseases[MeSH Terms] |
| #27cardiovascular diseases[Title/Abstract] |
| #28High Blood Pressures[MeSH Terms] |
| #29High Blood Pressures[Title/Abstract] |
| #30High Blood Pressure[MeSH Terms] |
| #31High Blood Pressure[Title/Abstract] |
| #32Blood Pressures, High[MeSH Terms] |
| #33Blood Pressures, High[Title/Abstract] |
| #34Blood Pressure, High[MeSH Terms] |
| #35Blood Pressure, High[Title/Abstract] |
| #36blood pressure[MeSH Terms] |
| #37blood pressure[Title/Abstract] |
| #38blood pressures[MeSH Terms] |
| #39blood pressures[Title/Abstract] |
| #40arterial pressure[MeSH Terms] |
| #41arterial pressure[Title/Abstract] |
| #42hypotension[MeSH Terms] |
| #43hypotension[Title/Abstract] |
| #44nmotension[MeSH Terms] |
| #45nmotension[Title/Abstract] |
| #46hypertensive[MeSH Terms] |
| #47hypertensive[Title/Abstract] |
| #48systolic pressure[MeSH Terms] |
| #49systolic pressure[Title/Abstract] |
| #50diastolic pressure[MeSH Terms] |
| #51diastolic pressure[Title/Abstract] |
| #52pulse pressure[MeSH Terms] |
| #53pulse pressure[Title/Abstract] |
| #54venous pressure[MeSH Terms] |
| #55venous pressure[Title/Abstract] |
| #56pressure monit[MeSH Terms] |
| #57pressure monit[Title/Abstract] |
| #58pre hypertension[MeSH Terms] |
| #59pre hypertension[Title/Abstract] |
| #60bp response[MeSH Terms] |
| #61bp response[Title/Abstract] |
| #62bp reduction[MeSH Terms] |
| #63bp reduction[Title/Abstract] |
| #64bp monit[MeSH Terms] |
| #65bp monit[Title/Abstract] |
| #66bp decrease[MeSH Terms] |
| #67bp decrease[Title/Abstract] |
| #68bp monits[MeSH Terms] |
| #69bp monits[Title/Abstract] |
| #70bp measurement[MeSH Terms] |
| #71bp measurement[Title/Abstract] |
| #72 #22 OR #23 OR #24 OR #25 OR #26 OR #27 OR #28 OR #29 OR #30 OR #31 OR #31 OR #32 OR #33 OR #34 OR #35 OR #36 OR #37 OR #38 OR #39 OR #40 OR #41 OR #42 OR #43 OR #44 OR #45 OR #46 OR #47 OR #48 OR #49 OR #50 OR #51 OR #52 OR #53 OR #54 OR #55 OR #56 OR #57 OR # 58 OR #59 OR # 60 OR # 61 # OR #62 OR # 63 OR #64 OR #65 OR # 66 OR # 67 OR #68 OR #69 OR #70 OR #71 |
| #73 randomized controlled trial[Publication Type] |
| #74 randomized[Title/Abstract] |
| #75 placebo[Title/Abstract] |
| #76 Controlled Clinical Trial[Publication Type] |
| #77 #73 OR #74 OR #75 OR #76 |
| #78 #21 AND #72 AND #77 |
